# Automatically Identifying Twitter Users for Interventions to Support Dementia Family Caregivers: Annotated Data Set and Benchmark Classification Models

**DOI:** 10.2196/39547

**Published:** 2022-09-16

**Authors:** Ari Z Klein, Arjun Magge, Karen O'Connor, Graciela Gonzalez-Hernandez

**Affiliations:** 1 Department of Biostatistics, Epidemiology, and Informatics Perelman School of Medicine University of Pennsylvania Philadelphia, PA United States; 2 Department of Computational Biomedicine Cedars-Sinai Medical Center Los Angeles, CA United States

**Keywords:** natural language processing, social media, data mining, dementia, Alzheimer disease, caregivers

## Abstract

**Background:**

More than 6 million people in the United States have Alzheimer disease and related dementias, receiving help from more than 11 million family or other informal caregivers. A range of traditional interventions has been developed to support family caregivers; however, most of them have not been implemented in practice and remain largely inaccessible. While recent studies have shown that family caregivers of people with dementia use Twitter to discuss their experiences, methods have not been developed to enable the use of Twitter for interventions.

**Objective:**

The objective of this study is to develop an annotated data set and benchmark classification models for automatically identifying a cohort of Twitter users who have a family member with dementia.

**Methods:**

Between May 4 and May 20, 2021, we collected 10,733 tweets, posted by 8846 users, that mention a dementia-related keyword, a linguistic marker that potentially indicates a diagnosis, and a select familial relationship. Three annotators annotated 1 random tweet per user to distinguish those that indicate having a family member with dementia from those that do not. Interannotator agreement was 0.82 (Fleiss kappa). We used the annotated tweets to train and evaluate support vector machine and deep neural network classifiers. To assess the scalability of our approach, we then deployed automatic classification on unlabeled tweets that were continuously collected between May 4, 2021, and March 9, 2022.

**Results:**

A deep neural network classifier based on a BERT (bidirectional encoder representations from transformers) model pretrained on tweets achieved the highest *F*_1_-score of 0.962 (precision=0.946 and recall=0.979) for the class of tweets indicating that the user has a family member with dementia. The classifier detected 128,838 tweets that indicate having a family member with dementia, posted by 74,290 users between May 4, 2021, and March 9, 2022—that is, approximately 7500 users per month.

**Conclusions:**

Our annotated data set can be used to automatically identify Twitter users who have a family member with dementia, enabling the use of Twitter on a large scale to not only explore family caregivers’ experiences but also directly target interventions at these users.

## Introduction

More than 6 million people in the United States have Alzheimer disease and related dementias, and the burden is projected to double by 2060 [1]. Alzheimer disease is the sixth leading cause of death in the United States [2], and only 8% of people with dementia do not receive help from family members or other informal care providers [3], amounting to more than 11 million family or other unpaid caregivers in 2020 [4]. Caregivers of people with dementia are impacted physically, cognitively, socially, mentally, and financially. For instance, compared with noncaregivers, they are more vulnerable to disease due to chronic stress [5] and have lower durations and quality of sleep [6]. Compared with non–dementia caregivers, they are more likely to experience a decline in cognition [7] and social network size [8]. They are also more likely to experience depression compared with noncaregivers [9] and non–dementia caregivers [10], and depressive symptoms in dementia caregivers are associated with increased health care use and costs [11]. In addition to the increased costs of their personal health care, family caregivers of people with dementia pay for much of the recipient’s total care costs, with the costs being significantly higher for people with dementia than without dementia [12].

A range of traditional interventions has been developed to support family caregivers of people with dementia [13]; however, most of them have not been implemented in practice and remain largely inaccessible [14]. Recent systematic reviews have concluded that internet-based interventions are valued by family caregivers of people with dementia for their easy access [15] and can have beneficial effects on caregivers’ health [16]. While recent studies [17-23] have shown that family caregivers of people with dementia use Twitter to discuss their experiences, to the best of our knowledge, methods have not been developed to enable the use of Twitter as a platform for internet-based interventions. Given that nearly 1 of every 4 adults in the United States uses Twitter [24], Twitter may present a novel opportunity to reach family caregivers on a large scale, such as through user-targeted advertisements providing information about dementia, caregiving, resources, or services. The objective of this study was to develop an annotated data set and benchmark classification models for automatically identifying a cohort of Twitter users who have a family member with dementia.

## Methods

### Ethical Considerations

The data used in this study were collected in accordance with the Twitter Terms of Service. The Institutional Review Board of the University of Pennsylvania reviewed this study (protocol number: 828972) and deemed it exempt human subjects research under 45 CFR §46.101(b)(4) for publicly available data sources.

### Data Collection and Annotation

Between May 4 and May 20, 2021, we collected 67,060 publicly available tweets from the Twitter streaming application programming interface (API) that are in English, are not retweets, and include both a dementia-related keyword (eg, *dementia*, *youngdementia*, *#yod*, *#ftd*, *alzheimer’s*, *alz*, *alzheimersdisease*, *mild cognitive impairment*) and a linguistic marker that potentially indicates a diagnosis (eg, *diagnosed*, *diagnosis*, *has*, *got*, *developed*, *with*, *from*). The full list of API search terms is available in Multimedia Appendix 1. We then searched these tweets for references to select familial relationships (Multimedia Appendix 2), identifying 10,733 (16%) of the 67,060 tweets. We randomly sampled 1 tweet per user—8846 (82%) of the 10,733 tweets—and developed annotation guidelines (Multimedia Appendix 3) to help 3 annotators distinguish tweets that indicate having a family member with dementia from those that do not. Among the 8846 annotated tweets, 8346 (94%) were dual annotated, and 500 (6%) were annotated by all 3 annotators. Interannotator agreement, based on the 500 tweets annotated by all 3 annotators, was 0.82 (Fleiss kappa). Upon resolving the disagreements, it was determined that 5946 (67%) of the tweets indicate that the user has a family member with dementia, and 2900 (33%) of the tweets do not.

### Automatic Classification

We performed benchmark supervised machine learning experiments to assess the utility of the annotated data set for automatically identifying Twitter users who have a family member with dementia. For the classifiers, we used the LibSVM [25] implementation of support vector machine (SVM) in Weka and SVM and 6 deep neural network classifiers based on BERT (bidirectional encoder representations from transformers): the BERT-Base-Uncased [26], DistilBERT-Base-Uncased [27], RoBERTa-Large [28], BioBERT-Large-Cased [29], Bio+ClinicalBERT [30], and BERTweet-Large [31] pretrained models in the *Flair* Python library. We split the 8846 tweets into 80% (7077 tweets) and 20% (1769 tweets) random sets as training data (Multimedia Appendix 4) and held-out test data, respectively, stratified based on the distribution of the binary annotated classes. For the SVM classifier, we preprocessed the tweets by normalizing URLs, usernames, digits, and keywords related to dementia (Multimedia Appendix 1) and familial relationships (Multimedia Appendix 2), removing nonalphanumeric characters and extra spaces, and lowercasing and stemming [32] the text. We used the Weka NGram Tokenizer to extract n-grams (n=1-3) as features in a bag-of-words representation. We used the radial basis function kernel and set the *cost* at *c*=32. For the BERT-based classifiers, we preprocessed the tweets by normalizing URLs and usernames and lowercasing the text. For training, we used stochastic gradient descent optimization, a batch size of 8, 15 epochs, and a learning rate of 0.001. During training, we fine-tuned all layers of the transformer model with our annotated tweets. To optimize performance, the model was evaluated after each epoch on a 5% split of the training set. To assess the scalability of our approach, we then deployed automatic classification on 198,674 unlabeled tweets, posted by 119,640 users, that were continuously collected from the Twitter streaming API (Multimedia Appendix 1) between May 4, 2021, and March 9, 2022, and mentioned a select familial relationship (Multimedia Appendix 2).

## Results

Table 1 presents the precision, recall, and *F*_1_-scores of SVM and 6 deep neural network classifiers for the class of tweets indicating that the user has a family member with dementia, evaluated on a held-out test set of 1769 (20%) of the 8846 manually annotated tweets. The classifier based on a model pretrained on tweets (BERTweet-Large) achieved the highest *F*_1_-score: 0.962 (precision=0.946 and recall=0.979). When deployed on 198,674 unlabeled tweets, posted by 119,640 users, between May 4, 2021, and March 9, 2022, the BERTweet classifier detected 128,838 tweets indicating that the user has a family member with dementia, posted by 74,290 users—that is, approximately 7500 users per month.

Table 2 presents examples of false positives and false negatives of the BERTweet classifier in the test set. Among the 68 false positives, 36 (47%) refer to people with dementia who are not or may not be select family members (Tweet 1), 8 (12%) report that a family member has a condition other than dementia (Tweet 2), and 5 (7%) merely speculate that a family member has dementia (Tweet 3). Another 8 (12%) of the 68 false positives were a result of manual annotation errors. Among the 25 false negatives, 14 (56%) use deixis or anaphora, requiring additional context in the tweet to understand that a non–first person determiner (eg, “their” in Tweet 4) actually refers to the user, or that a personal pronoun (eg, “she” in Tweet 5) refers to a select family member with dementia. Furthermore, 12 (86%) of these 14 tweets also include references to people who are not family members or do not have dementia. Another 4 (16%) of the 25 false negatives were a result of manual annotation errors.

**Table 1:** Precision, recall, and *F*_1_-scores of classifiers for detecting tweets indicating that the user has a family member with dementia.

Classifier	Precision	Recall	*F*_1_-score
SVM^a^	0.884	0.939	0.910
BERT^b^-Base-Uncased	0.924	0.954	0.938
DistilBERT-Base-Uncased	0.930	0.942	0.936
RoBERTa-Large	0.918	0.982	0.949
BioBERT-Large-Cased	0.907	0.978	0.941
Bio+ClinicalBERT	0.903	0.958	0.930
BERTweet-Large	0.946	0.979	0.962

^a^SVM: support vector machine.

^b^BERT: bidirectional encoder representations from transformers.

**Table 2:** Sample false positives and false negatives of a BERTweet classifier for detecting tweets indicating that the user has a select family member with dementia.

Tweet number	Tweet	Actual	Predicted
1	Evelyn has dementia, I know. But when she asked me today how my dad was doing... it still hurt.	–	+
2	We really don't have a clue about what causes Alzheimer's. We don't have a clue about Parkinson's, which is what got my dad, either.	–	+
3	I just listened to the Everywhere at The End of Time, by The Caretaker, and thought about my grandmother. The songs are about dementia, something my grandma wasn't clearly diagnosed with, but it hit hard.	–	+
4	If someone tells u their parent has Alzheimer's please don’t say your grandparent or great aunt did too. I appreciate that u can relate to the experience but it is so different. Tell me a different time.	+	–
5	I have a family member who is vulnerable and two children in their late 20s. I didn’t want to risk passing virus to her or from her to my family member. My sister made a bubble with her and her carers. She has dementia so she probably hasn’t missed me!	+	–

## Discussion

### Principal Findings

The benchmark performance of automatic classification demonstrates that our annotated data set has utility for accurately identifying Twitter users who have a family member with dementia, and deploying automatic classification on unlabeled tweets demonstrates that a large cohort of users can be identified. Therefore, our annotated data set enables the use of Twitter to scale up accessible, internet-based interventions directly targeted at family caregivers of people with dementia. Because our approach involves identifying tweets that mention a familial relationship, it would also enable interventions to be tailored to the care recipient.

### Limitations

Our approach to identifying family caregivers assumes that having “close” relatives with dementia would likely imply the users’ involvement in caregiving; however, the users identified in this study may not necessarily be caregivers or may have been caregivers but are no longer. We took this approach because we believe that limiting our identification of caregivers to users who explicitly state that they are providing ongoing care would underutilize the potential of Twitter for reaching caregivers on a large scale.

### Conclusions

This paper presented an annotated data set and benchmark classification models for automatically identifying Twitter users who have a family member with dementia, enabling the use of Twitter on a large scale to not only explore family caregivers’ experiences among their tweets but also directly target interventions at these users.

## Appendix

Multimedia Appendix 1 Twitter streaming application programming interface search terms.

Multimedia Appendix 2 Family member keywords.

Multimedia Appendix 3 Annotation guidelines.

Multimedia Appendix 4 Training data.

## Abbreviations

API: application programming interface
BERT: bidirectional encoder representations from transformers
SVM: support vector machine
