# Supplementary material for: Automatically Identifying Twitter Users for Interventions to Support Dementia Family Caregivers: Annotated Data Set and Benchmark Classification Models
Source: JMIR Aging. 2022 Sep 16;5(3):e39547. doi: 10.2196/39547 (PMC9526111; doi:10.2196/39547)
Supplement: Multimedia Appendix 3 [file aging_v5i3e39547_app3.pdf]

Identifying Twitter Users Who Have a Family Member With Dementia: Annotation Guidelines

The following brief guidelines will help annotators distinguish tweets that indicate having a family member with dementia (annotated as “1”) from those that do not (annotated as “0”). For the purpose of this annotation task, a family member is a close relative defined as any one of the following relationships to the Twitter user: parent, grandparent, sibling, spouse, or child, including any of these relevant relationships through marriage.

Tweets should be labeled as “1” if they indicate that the user has a family member with dementia:

[1] My 87 y/o grandfather with brain cancer got it, my 85 y/o grandmother with dementia got it, my 62 y/o mother with stage 4 OCP got it, my 59 y/o step-father with type 2 diabetes got it.Quit pretending like you know anything

[2] I’ve see the same trend with my mother in law and a friend who both have dementia. One even walks like Slow Joe.

[3] Holy fucking shit this is bad @Apple. Bad. Very bad.And I live with my father who has dementia and was considering an Apple Watch to help us find him when he wanders. Guess I could spend 1/10 of the $$ and just put this in his shoes?

While [1], [2], and [3] explicitly state that the users have a family member with dementia, not all tweets may be as explicit. Tweets should still be labeled as “1” if the annotator can infer that the mention of dementia refers to the user’s family member:

[4] Sidenote on that, if someone tells u their parent has Alzheimer's please suppress every desire to say your grandparent or great aunt did too. I appreciate the connection and that u can relate to the experience but it is so unbelievably different. Tell me a different time.

[5] My father was on Aricept, it made his symptoms worse. I don't think you can reverse Alzheimer's. Maybe someday gene therapy will stop it in it's tracks.I am concerned that Alzheimer's it becoming cottage industry. Too much profit to cure.

While some of the above tweets are ambiguous about whether the family member is living or deceased, other tweets may more clearly indicate that the family member is deceased:

[6] It is awful. I truly feel for you all. My mother had alzheimers and found things like a simple room change in her care home upsetting.

[7] My grandpa (RIP) had Parkinson's with Lewy Body Dementia for years before he passed. Occasional moments of modest clarity, but mostly confused and immobile, with no clue what year it was.I see similar physical characteristics in this photo

[8] Lost my mom in 2020 also. She was 95 and had Alzheimers. It was her time, but Mother’s Day without her is harder than I thought it would be.

Nonetheless, annotators do not need to distinguish family members that are living from those that are deceased, so [6], [7], and [8] should still be labeled as “1” for the purpose of this annotation task.

Tweets should be labeled as “0” if they self-report having dementia or refer to a family member beyond those defined as a close relative:

```
[9]  THIS MOTHER'S DAY I WOULD LOVE TO HAVE YOUR SUPPORT IN MY FIGHT
     AGAINST ALZHEIMERS. I WAS DIAGNOSED AT 42. PLEASE GO TO.
     https://t.co/YWdUKbNQ3c Join my team, Bobbie Cavazos and Donate.
     THIS WOULD MAKE THE BEST MOTHER'S DAY GIFT, BESIDE BRINGING BACK THE
     SUPER TACOS.
[10] Saw my uncle yesterday my Dad's brother. He has dementia. He thought
     I was my mum. She was about my age the last time e would have seen
     her. So strong genes on the maternal side.
```

Similarly, tweets should be labeled as “0” if they indicate knowing or being a caregiver for a person with dementia but do not specify a family member:

```
[11] The Father is the most painfully good movie I’ve seen in a long time.
     Anyone who’s experienced dementia firsthand is going to be
     uncomfortably at home with it; Anthony Hopkins’s performance is
     impeccable.
[12] As someone who cared for a dementia sufferer for a few years, maybe
     her daughter should have taken better care of her mom.
```

Tweets should also be labeled as “0” if they refer to a family member defined as a close relative but indicate that dementia is merely suspected:

```
[13] I think my mom is showing early signs of dementia, and I’ve been heart
     broken about it for the past month. Nothing matters right now than to
     be spending time with my parents, top priority.
```

In general, tweets should be labeled as “0” if they quote what was said by others:

```
[14] 'Initially we didn’t have any awareness of Alzheimer’s. It took a
     few years for Ba (we call her Ba – Gujarati for ‘grandma’) to get a
     diagnosis. At first we noticed changes in her behaviour and overall
     mood.' 1/#CureTheCareSystem #DAW2021
[15] Tangles: A Story About Alzheimer’s, My Mother, And Me
     https://t.co/gvIAFfzToW
```

In [14], the report of dementia is explicitly wrapped in quotation marks. In [15], the report of dementia appears to be in the title of a linked article being shared by the user.
